# Effect of Fatigue and Item Order On Students’ Performance in Multiple-choice Based Examinations: A Progress Test-Based Study

**DOI:** 10.1007/s40670-026-02883-5

**Published:** 2026-09-09

**Authors:** Pedro Tadao Hamamoto Filho, Laurens Bremers, Alba Regina de Abreu Lima, Leandro Arthur Diehl, Maria Cristina de Andrade, Maria de Lourdes Marmorato Botta Hafner, Miriam Hashimoto, Marcos Antônio Marton Filho, Samira Yarak, Júlio César Moriguti, Angélica Maria Bicudo, Andre Bremers

**Affiliations:** 1 Departamento de Neurociências e Saúde Mental, UNESP – Universidade Estadual Paulista, Campus de Botucatu, Distrito de Rubião Jr, s/n , Botucatu, SP Brazil 18.618-970; 2 Schoolyear BV, Den Bosch, Netherlands; 3 FAMERP – Faculdade de Medicina de São José do Rio Preto, São José do Rio Preto, Brazil; 4 UEL – Universidade Estadual de Londrina, Londrina, Brazil; 5 UNIFESP – Universidade Federal de São Paulo, São Paulo, Brazil; 6 FAMEMA – Faculdade de Medicina de Marília, Marília, Brazil; 7 USP – Universidade de São Paulo, Bauru, Brazil; 8 USP – Universidade de São Paulo, Ribeirão Preto, Brazil; 9 UNICAMP – Universidade Estadual de Campinas, Campinas, Brazil; 10 Radboud University Medical Center, Nijmegen, Netherlands

**Keywords:** Educational assessment, Progress test, Fatigue, Item list, Heterogeneity

## Abstract

**Introduction:**

Test length may influence performance through position-related effects, particularly toward the end of an examination due to fatigue. In computer-based tests with fixed navigation, item position may contribute to differences in performance that are not fully attributable to differences in knowledge.

**Methods:**

We investigated whether the performance of students from the 1st to the 6th undergraduate years on a 120-item multiple-choice Progress Test examination was influenced by item position and fatigue. The 120 items were first arranged in a randomized sequence across the different content areas. Once this sequence was established, it remained fixed, and the two groups completed it in opposite directions: Group 1 answered the items from 1 to 120, whereas Group 2 answered them from 120 to 1 and that this sequence. For comparisons between the groups, we used a pooled item-level analysis, calculating the proportion of correct answers for each item and its corresponding confidence interval. Subsequently, a random-effects proportional meta-analysis of the differences in item-level proportions was performed. Heterogeneity was assessed using the I² statistic.

**Results:**

Students in Group 1 outperformed those in Group 2 on the first batch of items, whereas the opposite pattern was observed for the second batch. Differences in heterogeneity decreased from the 1st to the 6th undergraduate years, suggesting a more stable response pattern among senior students than among junior students.

**Conclusions:**

Our findings suggest that fatigue may interfere with students’ performance on items presented toward the end of an examination. However, the effect was not strong enough to substantially affect overall scores. The different patterns observed across the 1st to 6th years suggest varying susceptibility to fatigue, indicating that fatigue may be an important consideration, particularly among less experienced students.

**Supplementary Information:**

The online version contains supplementary material available at https://doi.org/10.1007/s40670-026-02883-5.

## Introduction

A balance between psychometric recommendations, educational goals, and impact on student behavior should be maintained to design an educational assessment [1]. In an educational assessment, at least 80 items are required to achieve acceptable psychometric properties and the usual length ranges from 80 to 200 [2]. A high number of items may have a different impact on students’ behavior in the form of a fatigue effect in which students may be too tired to answer items at the end of the test. This behavior may have significant consequences, potentially leading to performance variability on later items that may not be fully explained by knowledge alone [3] and corrupts the formative value of the test. Understanding the fatigue effect may support the design of new educational assessment and lead to different considerations of pass/fail decisions and test sizes.

The Progress Test (PT) has been widely used for the assessment of undergraduate health sciences education and has been particularly useful in the context of programmatic assessment. It is based on the principle of longitudinal and repeated assessment of students’ functional knowledge [4, 5]. In practical terms, students from the first to the final undergraduate year take the same examination, which is designed primarily around the expected knowledge of a newly graduated professional. Periodically, all students take the same test at the same time, allowing their knowledge development to be assessed throughout their training. Each test administration follows a fixed content blueprint, although the specific items vary from one examination to another. In Brazil, where undergraduate medical education spans six years, with the initial years primarily devoted to basic sciences and the final years to clinical clerkships, the PT has been widely used across the country, mainly for formative purposes [6]. It is generally organized by consortia of medical schools and is administered once or twice a year.

In the PT context, students from the initial series are more likely to negatively appreciate PT benefits because they feel they cannot answer a test designed at the final-year level and feel angry that they are tested on things they have not been taught. The fatigue effect may pose even more negative appreciation on PT examinations. Brazilian medical students usually complain that PT examinations are too long [7]. These factors could influence student performance, with reports of students falling asleep at the end of the test [8–11].

Since the widespread use of computer-based online examinations, the discussion of item positions has gained more importance. Traditionally, in a paper assessment, students can move back and forward through test items, skipping and revisiting doubtful items [12]. However, in computer-based assessments, the item positions can be fixed.

Few papers have been published on similar topics, all in other fields than health care education. For example, Soureshjani [13] demonstrated that students who took a grammar test in the order of easy-to-difficult questions outperformed those who took the test from difficult-to-easy questions. One hypothesis for this finding is that, by the end of the exam, the latter students are more fatigued and mental exhaustion interferes with their cognitive abilities [13]. Accordingly, Otero and Alonso (2023) demonstrated that the sequence of questions in a cognitive-reflection test affects performance and that response times are longer for the first questions than for the last ones [14]. 

In the present study, we investigated whether students’ performance on the PT was influenced by item position and, consequentially, by the fatigue effect.

## Methods

This cross-sectional analytical observational study used data from an interinstitutional PT examination, i.e., the data from this study were all gathered in one concurrent (i.e., same year) administration of the PT.

### Settings

This inter-institutional PT is administered twice a year in ten public Brazilian medical schools (nine in São Paulo State and one in Paraná State). It comprises 120 multiple-choice questions with a single best answer (MCQ, items) divided into internal medicine (34 MCQ), pediatrics (22), surgery (22), obstetrics and gynecology (20), and public health (20). PT has a fixed blueprint for the content to be sampled across all examinations (i.e., the number of items on cardiology, neurology, mastology, etc., is fixed; however, for each test administration, the items of each specialty are different and original). This PT has been administered online since 2020, and students cannot go forward and back across the items; that is, a student cannot view an item again after marking it.

For this examination, the 120 items were first arranged in a randomized sequence across the different content areas, and this sequence was subsequently kept unchanged for the study. For example, the test was not organized into blocks of internal medicine, pediatrics, surgery, public health, and so on. Rather, items from different content areas were interspersed (e.g., a pediatrics item could be followed by a surgery item and then a public health item). Once this sequence had been established, it remained fixed, and two groups completed it in opposite directions: Group 1 answered the items from 1 to 120, whereas Group 2 answered them from 120 to 1. Students were allocated according to the last digit of their document number, whether odd (Group 1) or even (Group 2). This document number is random, and leads to no distinction between age, sex, or geographical origin.

The examination was administered on a midweek afternoon, and students were allowed to take a break at their own discretion during the 4-hour period allotted to complete the test.

### Data processing and analysis

The students’ answers were coded as 0 or 1 for incorrect or correct answers, respectively. Blank responses were counted as incorrect responses. We calculated differences between the two groups in the correct answer rates of each item. Next, we also performed a linear regression considering the items as independent variables (from 1 to 120) and the right-answer rate differences as dependent variables. We considered the intercept point of the linear regression with the *x* axis the point at which the difference rates changed, favoring one group over the other.

For comparisons between groups, we used a pooled analysis of the item approach [15]. Briefly, it involved calculating the rate of correct answers for each item, along with the corresponding 95% confidence interval (CI). Subsequently, a proportional meta-analysis of the item-rate differences and CI with random effects was performed. The meta-analysis revealed a statistically significant odds ratio when the final CI did not reach the value of 1. Finally, the meta-analysis provided an I^2^ value that referred to heterogeneity. High I^2^ values indicate a highly heterogeneous rate of correct answers across items between the groups, while low I^2^ values indicate more homogeneity for the observed CI.

We tested the average number of correct items between the two groups, considering the two halves of the exam set at 60 (middle of the 120 items) or at the intercept value, as previously described.

The Kolmogorov-Smirnov test was used to verify the data distribution. Normally distributed differences in averages were tested using Student’s T test and the Mann-Whitney U test for non-parametric data. Statistical analyses were performed using MedCalc for Windows, version 19.4 (MedCalc Software, Ostend, Belgium) and GraphPad Prism for MacBook, version 9.5.0 (GraphPad Software, Inc. San Diego, CA, USA). The statistical significance level was set at 5%.

## Results

We recorded answers from 3,985 students who were allocated to Group 1 (*n* = 1,835) and Group 2 (*n* = 2,150). Table 1 presents the number of students in each undergraduate year and group. A comparison of students’ scores on the entire exam showed no differences between the two groups in any of the undergraduate years.

**Table 1:** Number of students enrolled according to undergraduate year and allocation group

Year	Group1/Group2	Total
1st	305/322	627
2nd	264/352	616
3rd	308/390	698
4th	339/357	696
5th	317/364	681
6th	302/365	667

**Fig. 1:**
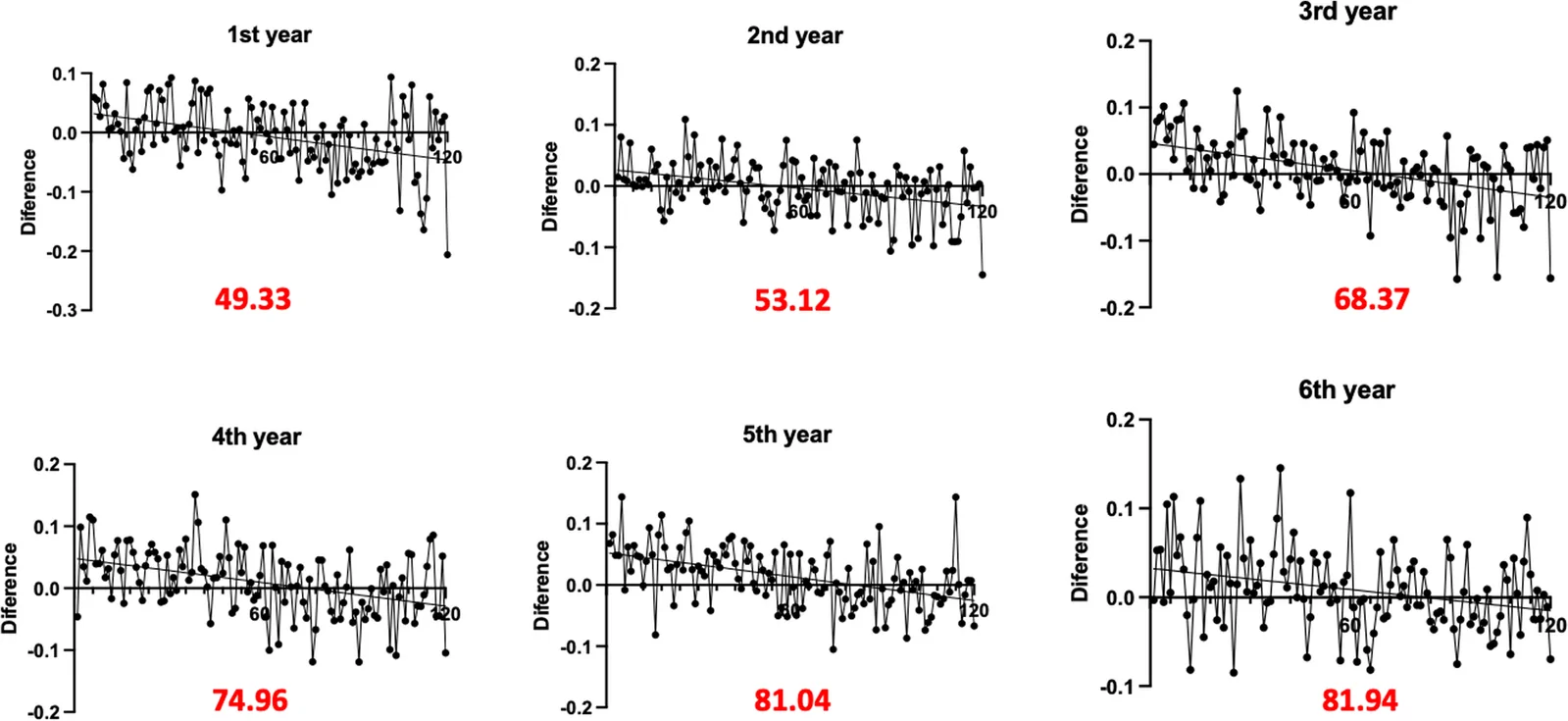
Differences between the two groups in the 120 items from the exam. *Y* axis refers to the difference in the rates of correct answers, with positive values pointing to the higher performance of group 1, and negative values to the higher performance of group 2. The *X* axis is the sequence of items, from 1 to 120. For all the undergraduate years, the regression line has a negative slope, which means that it begins with a better performance of group 1 on the first items and ends with a better performance of group 2 in the last items (located in the beginning of the exam for this group). The intercept values are in red at the bottom of the graphs

In general, students from Group 1 outperformed those from Group 2 on the first batch of items, with an inversion of the performance for the second batch, as Fig. 1 shows. The intercept values (at which the regression line crossed the *X* axis, i.e., the item from which the inversion between the groups inverted) varied from 49.33 (1st year) to 81.94 (6th year, increasing across training years). However, the overall mean scores of the students did not differ between the groups in any of the training years.

For the proportional meta-analysis of differences in the rate of items (Table 2, Supplemental Material), we divided the batch of items into two using two cutoff values: in the first one, the previously shown intercept value was considered, and in the second, the value was at 60 (i.e., the exact half of the exam). Students from group 1 outperformed in the first-batch items and underperformed in the second batch. These differences occurred in almost all undergraduate years and were statistically significant for the 5th and 6th year students (except for the second batch of items). When dividing the batch of items at 60, we observed the same pattern of out- and under-performance, with almost all differences reaching statistical significance, except for the second batch of items in the 3rd, 5th, and 6th years.

**Table 2:** Proportional metanalysis of differences between the groups in the correct answers for items

Year		Cutoff at intercept value		Cutoff at 60	
		OR (95% CI)	p-value	OR (95% CI)	p-value
**1st**	*First batch*	1.082 (1.031–1.139)	0.013	1.068 (1.010–1.129)	0.020
	*Second batch*	0.898 (0.842–0.958)	0.001	0.880 (0.820–0.945)	< 0.001
**2nd**	*First batch*	1.055 (1.003–1.108)	0.037	1.059 (1.009–1.110)	0.019
	*Second batch*	0.931 (0.883–0.982)	0.008	0.914 (0.866–0.965)	0.001
**3rd**	*First batch*	1.113 (1.060–1.168)	< 0.001	1.121 (1.066–1.179)	< 0.001
	*Second batch*	0.918 (0.855–0.986)	0.018	0.937 (0.877–1.001)	0.054
**4th**	*First batch*	1.125 (1.070–1.182)	< 0.001	1.166 (1.109–1.226)	< 0.001
	*Second batch*	0.924 (0.857–0.966)	0.038	0.935 (0.877–0.997)	0.040
**5th**	*First batch*	1.123 (1.068–1.180)	< 0.001	1.184 (1.121–1.251)	< 0.001
	*Second batch*	0.947 (0.878–1.021)	0.154	0.956 (0.904–1.012)	0.119
**6th**	*First batch*	1.086 (1.029–1.146)	0.003	1.132 (1.061–1.208)	< 0.001
	*Second batch*	0.957 (0.895–1.022)	0.190	0.963 (0.916–1.013)	0.147

CI: confidence interval, OR: odds ratio

Notes: Odds ratio (OR) values higher than 1 point to the outperformance of group 1, whereas OR values < 1 point to the outperformance of group 2. The intercept values were 49, 53, 68, 75, 81, and 82 for the 1 st to the 6th undergraduate years, respectively

**Fig. 2:**
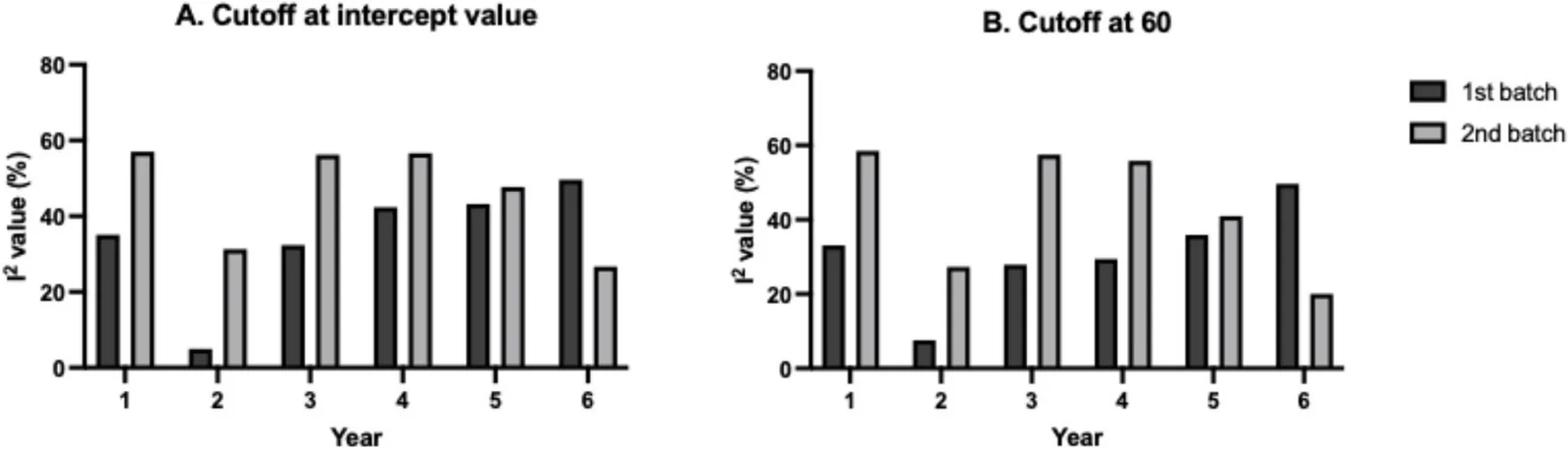
The heterogeneity values (I^2^) of the rates of correct answers of the items between groups 1 and 2. Higher I^2^ values suggest greater variability in the rates of correct answers between the groups. For both cutoff values (A – intercept, and B – 60), the second batch of items presented higher heterogeneity than the first batch for all undergraduate years (1 to 6)

The I^2^ values—the heterogeneity between the items’ rates—were higher for the second batch of items than for the first one in all undergraduate years, except for the 6th year. In the first batch, the I^2^ values increased from the 1 st to the 6th year – and the opposite was observed in the second batch: the I^2^ values decreased from the 1 st to the 6th year. Only the 2nd undergraduate year showed an atypical pattern (Fig. 2).

**Fig. 3:**
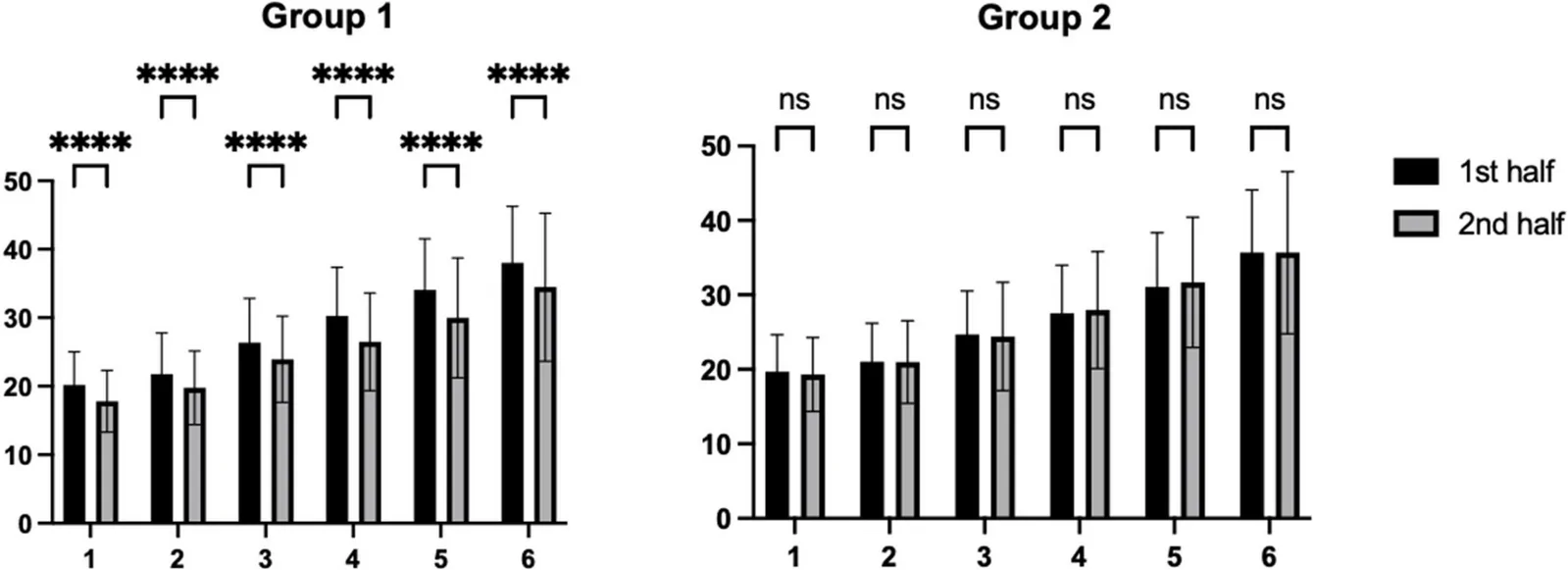
Comparisons of the mean of correct answers between the two halves of the exam. The first group had significantly higher scores in the first half of the exam than in the second one. Conversely, differences between the two halves were not significant for group 2

When the mean of correct answers between the two halves of the test was compared, Group 1 was found to have significantly lower scores in the second half than in the first half; this effect was observed for all undergraduate years. However, Group (2) maintained the same performance in the two halves of the test; that is, the mean of correct answers did not differ between the two batches of items, even though the overall mean scores were not different between the groups (Fig. 3).

## Discussion

That a higher accuracy of the assessment results from an increasing the number of items in an exam is empirically accepted. The accuracy can be measured through the internal consistency of the test with reliability indices. Two commonly used tools, Cronbach’s alpha and the Kuder-Richardson formula, depend on the number of items [16]. 

However, extensive examinations may discourage students, especially those who are unfamiliar with the subject being assessed [17]. This is the case for PT exams administered to students at the initial training levels, who report getting asleep and distressed upon concluding that they do not know much of the assessed content [9]. In a Brazilian study of medical students’ perceptions of the PT, more than 40% of students reported that PT was too long and could benefit from a reduction in the number of items [7].

Therefore, end-of-test effects (including potential fatigue and disengagement) may negatively affect performance on later-positioned items. We observed lower performance on later-positioned items, consistent with end-of-test effects, and that the fatigue effect could be reduced with a more amenable sequence of items. Nevertheless, these observations were not homogeneous for all undergraduate levels, and students in their initial years seemed to be more susceptible to these factors. Moreover, the effect of fatigue did not affect students equally.

Because we did not directly measure perceived fatigue (e.g., self-report) or process indicators (e.g., response times), we acknowledge that alternative explanations such as motivation, disengagement, or test-taking strategies could have interfered with our results. It is possible that students in the earlier years were less motivated and engaged in completing the test, whereas senior students, being more familiar with the test format, may have developed test-taking strategies to better manage their time. Future studies could build on our findings and incorporate designs that allow these potential confounders to be better controlled and the independent effect of fatigue to be more accurately assessed.

Jensen et al. did not find lower performance in lengthier exams owing to fatigue, despite the subjective perception of fatigue among undergraduate biology students [17]. Other studies evaluating test length and cognitive fatigue using the Scholastic Assessment Test have reported increased perceived fatigue among students who spent increasing time on tasks. However, fatigue did not have a significant effect on students’ performance or differential-item functioning [18–20]. All those studies were conducted using examinations designed at the level of the examinees. Conversely, the PT is designed at the level of a just-graduated student, but administered to all undergraduate students. Ackerman and Kanfer hypothesized that differences in students’ personality, interest, and motivation traits may play a more significant role in determining perceived fatigue than test-length situations [20]. In the case of PT, students’ interest and motivation from the initial and final years are probably different, which would add to differences in their perceived fatigue and willingness to answer the final questions [21]. This phenomenon has been described as “test-taking effort,” defined as “the extent to which an examinee gives his or her best effort” [22], and has been shown to influence student performance, particularly in low-stakes assessments such as the PT [23].

We also observed the move of the intercept value to the right (e.g., to more advanced items of the sequence) across years, beginning at 49 in the 1 st year and reaching 81 in the 6th year. The intercept value was assumed to be 60 if the fatigue effect was equal between the two groups. This relevant finding has two implications. First, the sequence of items favored one group over the other. Group 1 scored higher in the first half than the second half of the test (for all undergraduate years). This effect was not observed in Group 2. Therefore, the sequence of items may favor students’ performance, which could be explained by the spreading activation theory: recalling information triggers the activation of related concepts in memory, creating a chain reaction that results in the retrieval of a network of interconnected information [24]. Second, if the X-intercept moves to the right across the years, this may suggest that the fatigue effect is more pronounced among junior students, whereas senior students may be more resilient to fatigue, as suggested by the heterogeneity analysis. The difference in heterogeneity (I²) between the first and second batches of items decreased from the 1 st to the 6th undergraduate year, suggesting a more stable pattern of responses among senior students than among junior students. The heterogeneity analysis also supports the hypothesis that item sequence may affect students’ performance on individual items, as heterogeneity was higher for the second batch of items than for the first in all undergraduate years except the 6th year.

The mean global scores of the two experimental groups did not differ significantly. However, Group 2 showed no statistically significant difference in performance between the first and second batches of items, whereas Group 1 showed a decrease in performance in the second batch. Although we cannot establish a definitive explanation for this finding, one possible interpretation is that the sequence of items may have influenced performance differently in the two groups. For example, information presented in earlier items may have helped students answer subsequent items or, alternatively, may have interfered with their responses to subsequent items. Thus, although the overall performance of the two groups was similar, the different patterns observed within the groups suggest that the sequence in which items are presented may deserve greater attention and further investigation in future studies. These findings have practical implications for adaptive tests based on previously calibrated items. The position of an item early in a calibrating test may lead to an underestimation of its difficulty and, conversely, to an overestimation of the difficulty of items positioned late in the test. Recent data from the Trends in International Mathematics and Science Study have shown a higher difficulty of items blocked at the end of the exam [25, 26]. 

Nowadays, medical education faces the challenge of moving beyond reliability to greater accuracy, which involves students’ scores and their distance to the cut-off score. Thus, not only are reliability indices relied on for information on exam length [27]. Artificial increases in exam length to increase reliability coefficients based solely on internal consistency are not adequate and do not guarantee construct validity. The use of high-quality items preferably pretested in reproducible settings are more important. For high-stakes examinations, high reliability is necessary to provide evidence to support the measure. However, for assessments with lower consequences, such as formative or summative classroom-type assessments, high reliability is not sufficient [28]. In this context, a shorter exam that will not threaten the items’ properties with the fatigue effect can be acceptable. Our findings suggest that improved reliability should not be reached by increasing the length of test; increasing the number of tests may provide a sensible alternative.

Some limitations should be noted in our study. First, we did not directly measure fatigue (e.g., perceived fatigue scales) or behavioral process indicators (e.g., response times), which limits causal attribution. Second, position-related differences may also reflect motivation, disengagement, or test-taking strategies, particularly in early-year students taking a graduation-level Progress Test. Third, although the study is multi-institutional, the findings reflect the Brazilian Progress Test context and the specific computer-based platform constraints.

Nevertheless, it is worth emphasizing that the setting of a progress test differs from that of conventional examinations, as a larger proportion of questions cannot be answered based on prior knowledge, particularly in the earlier years of training. In this context, our findings are novel in that they provide a basis for estimating the maximum feasible length of such a progress test. Furthermore, the specific format of the progress test used here should be considered an optimal setting for conducting this experiment rather than a limitation to its external validity: presenting items in a randomized order or varying them across individuals would have hindered interpretation.

In conclusion, our findings suggest that the fatigue effect can interfere with students’ performance on the last-allocated items. However, these effects were not large enough to meaningfully change overall scores. Part of the fatigue effect can be reduced with a more amenable sequence of items. During their training, students may become more resilient to fatigue; that is, senior students may be less susceptible to fatigue during lengthy examinations. Future studies should build on our findings by incorporating additional variables, such as personal characteristics and the time spent completing the test.

## Supplementary Information

Below is the link to the electronic supplementary material.

Supplementary Material 1 (DOCX 113 KB)

## Data Availability

Datasets are available from the corresponding author on reasonable request.
