# Supplementary material for: Effect of Fatigue and Item Order On Students’ Performance in Multiple-choice Based Examinations: A Progress Test-Based Study
Source: Med Sci Educ. 2026 Sep 9;36(6):2897–904. doi: 10.1007/s40670-026-02883-5 (PMC13759997; doi:10.1007/s40670-026-02883-5)
Supplement: Supplementary file 1 [file 40670_2026_2883_MOESM1_ESM.docx]

A B

Supplemental material. Graphical representation of the odds ratios for correct responses to items in the first and second batches, according to training level. * indicates that the corresponding value is not significantly different from 1, i.e., the confidence interval crosses 1, meaning a p > 0.05.
